# Optical genome mapping uncovers disease-defining variants in an adult T-lymphoblastic leukemia and impacts prognosis

**DOI:** 10.1186/s13039-026-00770-7

**Published:** 2026-05-24

**Authors:** Amanda M. Maxfield, Michelle A. Bickford, Kyle A. Tonseth, Jing Bao, Farzana Murad, Devon N. Wilson, Lauren M. Wainman, John M. Hill, Liam L. Donnelly, Prabhjot Kaur, Laura J. Tafe, Jeremiah X. Karrs, Wahab A. Khan

**Affiliations:** 1 Geisel School of Medicine at Dartmouth, Hanover, NH USA; 2 Department of Pathology and Laboratory Medicine Dartmouth Health, Lebanon, NH USA; 3 Department of Hematology/Oncology Dartmouth Health, Lebanon, NH USA

**Keywords:** Optical genome mapping, Acute lymphoblastic leukemia, Risk stratification, Structural variants, Next-generation sequencing, Chromosomal rearrangements, *NOTCH1* alteration, *FBXW7* pathway

## Abstract

**Background:**

Adult T-lymphoblastic leukemia often harbors cryptic structural variants that remain undetected by standard cytogenetic and targeted molecular testing, limiting precise risk stratification and therapeutic planning. This case is notable for the use of optical genome mapping (OGM) to uncover multiple disease-defining and likely oncogenic genomic alterations in an adult patient with newly diagnosed T-lymphoblastic leukemia. The report highlights how comprehensive structural variant profiling can refine prognosis and identify clinically meaningful aberrations that would otherwise be missed in routine practice.

**Case presentation:**

A 29-year-old man presented with fever, chills, dyspnea, headache, petechiae, and was found to have pancytopenia. Bone marrow biopsy showed a hypercellular marrow with approximately 95% involvement by T-lymphoblasts and residual trilineage hematopoiesis. Flow cytometry demonstrated a T-lymphoblastic immunophenotype with expression of cytoplasmic CD3, CD7, terminal deoxynucleotidyl transferase (TdT), absent surface CD3, CD20, CD34, CD117, and myeloperoxidase. Conventional workup with fluorescence in situ hybridization (FISH) initially detected a *CDKN2A* loss but did not fully define prognosis. OGM revealed a deletion involving *FBXW7*, a *TLX3*::*BCL11B* fusion (subsequently confirmed by FISH), a hemizygous deletion involving *PHF6*, a duplication involving *MYB*, and a deletion involving *CDKN2A*, while next-generation sequencing identified a *NOTCH1* missense variant at approximately 50% variant allele fraction. A normal male karyotype was detected at post-induction staging. The patient was counseled on the overall favorable prognostic markers and started on induction therapy using the AALL1231 regimen for T‑lymphoblastic leukemia, including protocol-directed incorporation of bortezomib.

**Conclusions:**

OGM revealed multiple clinically relevant structural variants and partner genes impacted in adult T-lymphoblastic leukemia that were not part of the standard cytogenetic workup, thereby improving genomic characterization and risk assessment. Identification of alterations involving *FBXW7*, *NOTCH1*, *TLX3*::*BCL11B*, *PHF6*, *MYB*, and *CDKN2A* provided a comprehensive genomic profile that informed counseling regarding prognosis and supported treatment selection. Integrating OGM into routine evaluation of myeloid and lymphoid neoplasms has the potential to streamline diagnostic workflows and ensure that disease-defining aberrations critical for diagnosis, prognosis, and targeted therapy selection are not overlooked.

**Supplementary Information:**

The online version contains supplementary material available at 10.1186/s13039-026-00770-7.

## Background

Acute lymphoblastic leukemia (ALL) is a malignancy that originates from the uncontrolled proliferation of B or T lymphoid progenitor cells (i.e. B-ALL, T-ALL) [1]. ALL is most common during childhood, with its peak incidence in 0–9-year-olds, although there is a significant proportion of cases in young adults and patients above 50 years old, with the 5-year survival rate for 50–54-year-olds being < 30% [2, 3]. The classification of B- and T-ALL subtypes is valuable in determining one’s prognosis but has proven difficult due to the heterogeneity of genetic abnormalities associated with ALL. Additionally, multiple molecular and cytogenomic technologies are required to discover somatic variants, particularly structural rearrangements that are often present in these complex ALL cases.

Established DNA-based technologies such as optical genome mapping (OGM) have expanded capabilities to provide an unbiased assessment of structural variants (SVs), thus improving the classification of ALL subtypes and consolidating standard-of-care cytogenetic testing modalities [4–7]. The current National Cancer Comprehensive Network (NCCN)-defined guidelines for ALL and other hematolymphoid malignancies have expanded disease-defining targets [8, 9]. This has, in turn, poised technologies such as OGM to capture the spectrum of these findings. Herein, we present a case of a T-ALL patient in which multiple genetic abnormalities were identified by OGM, which was paramount in determining the patient’s prognosis.

## Case presentation

**Fig. 1:**
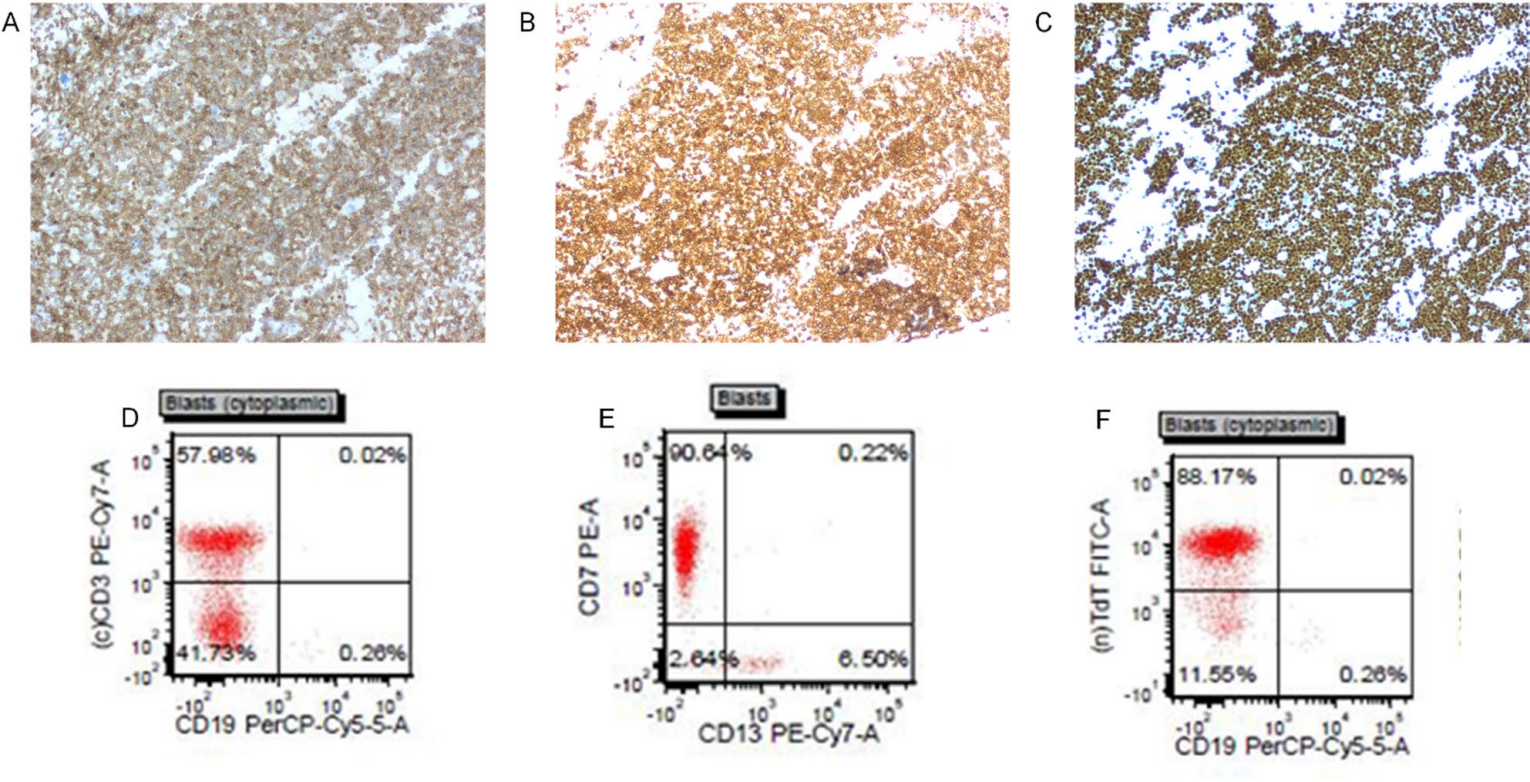
**(A-C)** Immunohistochemical workup positive for T-cell markers CD1a, CD3, and TdT, respectively (left to right). **(D-E).** Flow cytometry analysis depicting positive gate profile for CD3, CD7, and TdT nuclear markers, respectively (left to right)

The patient is a 29-year-old male who presented with a 2-day history of fever, chills, dyspnea, headache, and petechiae. He was found to have pancytopenia on complete blood count and was admitted for further workup. Bone marrow aspiration showed markedly increased blasts with decreased granulopoiesis and erythropoiesis. A bone marrow biopsy demonstrated a hypercellular marrow extensively infiltrated by immature cells with fine, dispersed chromatin and distinct nucleoli. Trilineage hematopoiesis was present but markedly diminished. Immunohistochemical analysis of the bone marrow biopsy demonstrated T-cell markers positive for CD1a, CD3, and terminal deoxynucleotidyl transferase (TdT) (Fig. 1A-C), with CD20 being negative. Flow cytometric analysis of bone marrow aspirate confirmed the immunophenotype positive for a T-cell population (positive cytoplasmic CD3 (cCD3), CD7, and TdT-nuclear markers) (Fig. 1D-E). Surface CD3 was notably negative despite cCD3 positivity while CD20, CD34, CD117, and cytoplasmic myeloperoxidase were all negative. The immunophenotype was consistent with precursor T-lymphoblastic leukemia.

Standard cytogenetic evaluation by fluorescence in situ hybridization (FISH) was performed on 200 interphase nuclei derived from bone marrow cells and employed locus-specific and dual color with dual fusion probes following manufacturer instructions and institutional standard operating procedures. Metaphase karyotyping was not performed at initial work-up, as targeted T-ALL specific FISH and OGM were prioritized to enable sensitive detection of clinically relevant submicroscopic and structural alterations. A conventional karyotype was subsequently obtained post-induction to complement these findings. Further, at the time of this diagnostic work‑up, T-ALL FISH was performed at a reference laboratory. In particular, the *TLX3*/*BCL11B* FISH probe set was a laboratory‑developed dual‑color fusion probe targeting *TLX3* (5q35) and *BCL11B* (14q32). This was specifically a ‘home‑brew’ BAC‑based probe design that was labeled with spectrally distinct fluorophores. The broad genomic coverage of the flanking probes and their concordance with the OGM breakpoints that explained the atypical fusion is depicted below. Next-generation sequencing (NGS) was carried out using an exome backbone (Agilent v8) based hematologic malignancy panel on an Illumina NovaSeq system and variants were annotated (AUGMET) and classified according to AMP/ASCO guidelines in the context of known T-ALL–associated alterations [10]. OGM was performed on ultra-high molecular weight DNA extracted from bone marrow aspirate using the Bionano Prep SP-G2 Bone Marrow Aspirate DNA Isolation Kit (part nos. 80118, 80062). Fluorescent labeling was performed with the DLE-1 kit (catalog no. 80005) and molecules imaged on the Saphyr flow cell (chip design: G3.3). The imaged molecules were subsequently aligned to the GRCh38 human Reference Genome to enable genome-wide detection of structural variants. A molecule N50 (weighted median value) of 294.7 kilobase pairs, ~ 480x effective coverage depth, and a 93% map rate was achieved post mapping.

**Fig. 2:**
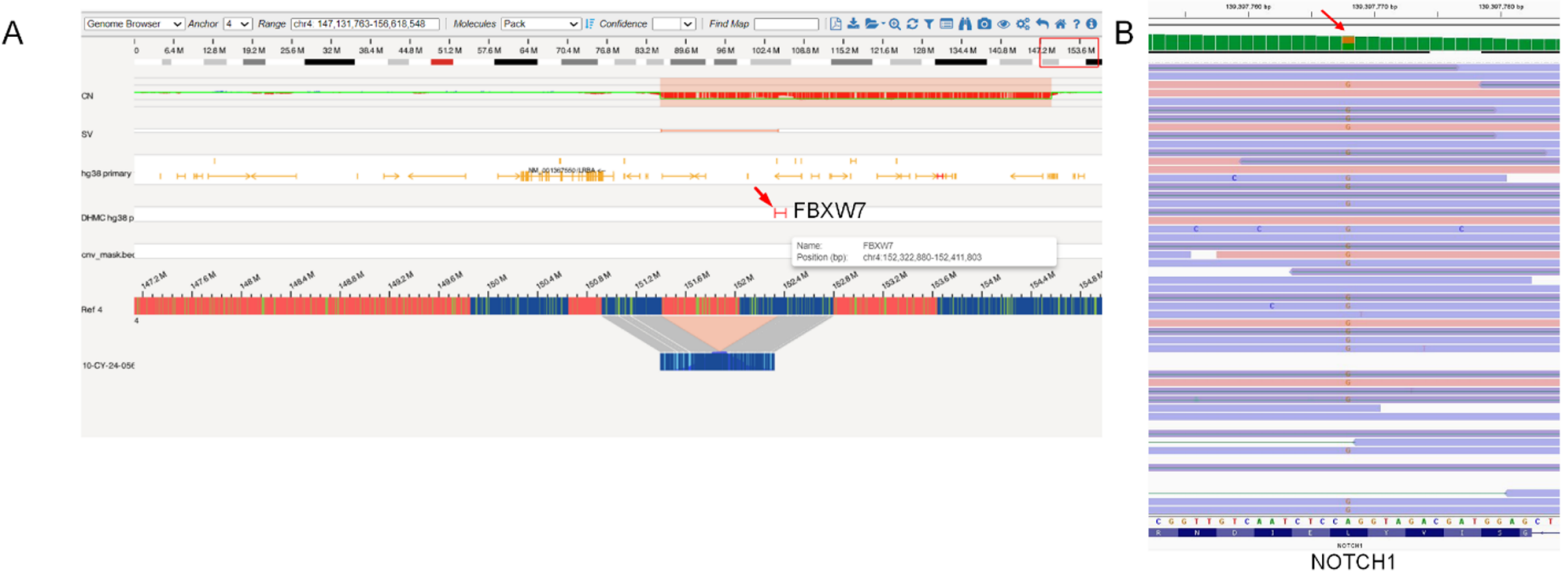
**(A)** Optical genome mapping detected a 3.170 Mb deletion (ogm[GRCh38] 4q31.3(151,393,045_154,563,349)x1) within chromosome 4q31.3 overlapping *FBXW7* (red arrow) with a variant allele fraction (VAF) of 0.435. **(B)** Integrated genome viewer shows sequence read pile from NGS data of a missense variant in *NOTCH1* at VAF of 0.503 (red arrow)

Risk stratification in T-ALL is determined using clinical markers such as age, white blood cell count, and disease classification, as well as *RAS/PTEN* and *NOTCH1*/*FBXW7* mutation status, such that *NOTCH1*/*FBXW7* variants are associated with a favorable prognosis in the absence of *RAS/PTEN* variants [8, 11]. In this context, OGM identified a 3.1 Mb deletion overlapping chromosome 4q31.3, including the entire *FBXW7* gene (Table 1; Fig. 2A). NGS complemented this result, identifying a *NOTCH1* (NM_017617.5) c.5033T > C p.(L1678P) variant within exon 27 involving its regulatory domain (Fig. 2B) [12]. NGS further confirmed the deletion, involving *FBXW7*, by our exome‑based copy number analysis (Additional file 1: Figure S1). The identification of a *NOTCH1*/*FBXW7* T-ALL-specific aberration (Fig. 2) demonstrates the utility of a combined genomic-scale OGM and NGS approach in detecting important markers of prognosis that would be otherwise missed by targeted or standard-of-care testing.

**Table 1:** Clinically significant variants detected by Optical Genome Mapping in T-ALL case, including the affected genes and their reported clinical significance

Variant	Gene(s) Affected	Clinical Significance
3.1 Mb deletion within 4q31.3	Deletion of *FBXW7*	Favorable prognosis in the absence of *RAS/PTEN* variants (8, 11)
1.085 Mb hemizygous deletion within Xq26.2	Deletion of *PHF6* exons 1–3	Favorable prognosis (13)
Insertional translocation involving 5q35 and 14q32	*TLX3:BCL11B* rearrangement	Recent data suggests favorable outcomes, although further investigation is needed (14–16)
197.1 kb duplication in 6q23.3	Duplication of *MYB*	Unknown
353.3 kb heterozygous deletion in 9p21.3	Deletion of *CDKN2A*	Poor prognosis (21, 23, 24)

**Fig. 3:**
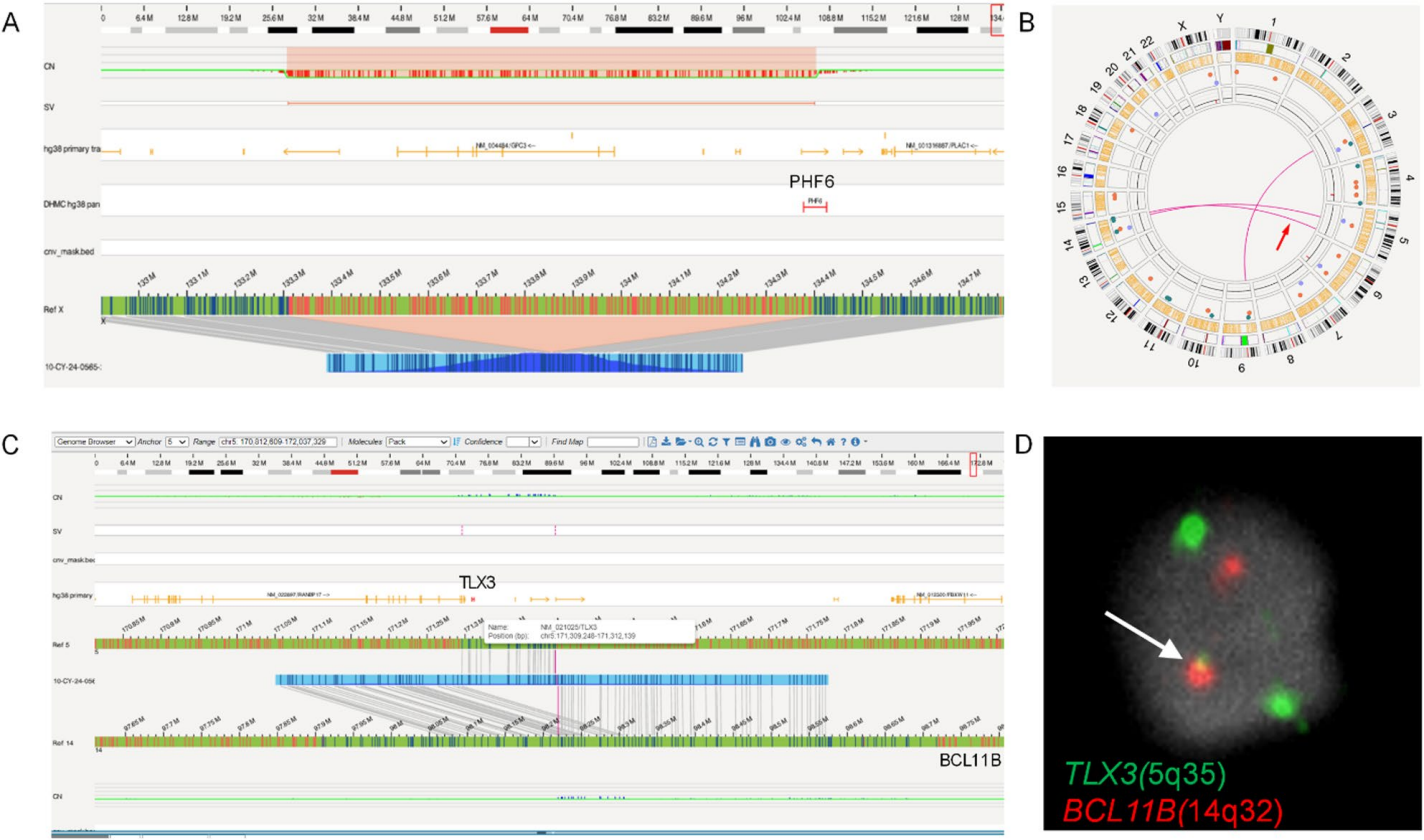
**(A)** Optical genome mapping detected a 1.085 Mb hemizygous deletion (ogm[GRCh38] Xq26.2(133,309,358_134,400,444)x1) in this male patient within chromosome Xq26.2 with a VAF of 0.910. **(B)** A circos plot for this T-ALL case highlighting an oncogenic rearrangement between chromosomes 5 and 14 is depicted (red arrow). **(C)** Zoom in view of the genome map shows this rearrangement to be an insertional translocation that involves transcription factor *TLX3* on 5q35 cytoband that is juxtaposed to *BCLL1B* regulatory region on 14q32 in T-ALL with a VAF of 0.530 (ogm[GRCh38] t(5;14)(q35.1;q32.2)(171296249;98306258 ~ 98305665). **(D)** Interphase FISH analysis confirms this rearrangement, demonstrating an atypical yet interpretable *TLX3*::*BCL11B* fusion signal with a dual‑color DNA FISH probe (white arrow), concordant with the optical genome mapping breakpoints. Nuclei is stained DAPI (inverse gray scale shown)

OGM detected additional SVs listed in ALL guidelines as being of known pathologic significance [9]. These include the deletion of tumor suppressor *PHF6* on chromosome Xq26.2 (Table 1; Fig. 3A) and a rare insertional translocation involving chromosomes 5 and 14, resulting in rearrangement of *TLX3* and *BCL11B* genes (Fig. 3B-C) with 105 supporting molecules that was subsequently corroborated by interphase FISH analysis in approximately 90% of nuclei (Fig. 3D). The deletion involving *PHF6* was confirmed by exome-based NGS (Additional file 1: Figure S2). Recent risk stratification has shown that *PHF6* variants are associated with favorable outcomes in adult T-ALL [13]. OGM also revealed a balanced t(3;9)(p12.3;p21.3) (Fig. 3B) without an obvious T‑ALL–associated or known leukemia gene impacted at the mapped breakpoints. This translocation between chromosomes 3 and 9 was subsequently classified as a structural variant of uncertain clinical significance. *TLX3* rearrangements have been identified in 10% of adult T-ALLs, with *BCL11B* being a commonly reported rearrangement partner [9]. In our case, we detected an insertional translocation that juxtaposed *TLX3* near the *BCL11B* enhancer regulatory region (Table 1; Fig. 3C). This cryptic rearrangement does not necessarily confer an inferior prognosis and has demonstrated an overall good response to venetoclax-based treatment [14]. Further, these results are interpreted in the context of a favorable mutational landscape associated with this alteration according to the International Consensus Classification (ICC) of ALL [15, 16].

**Fig. 4:**
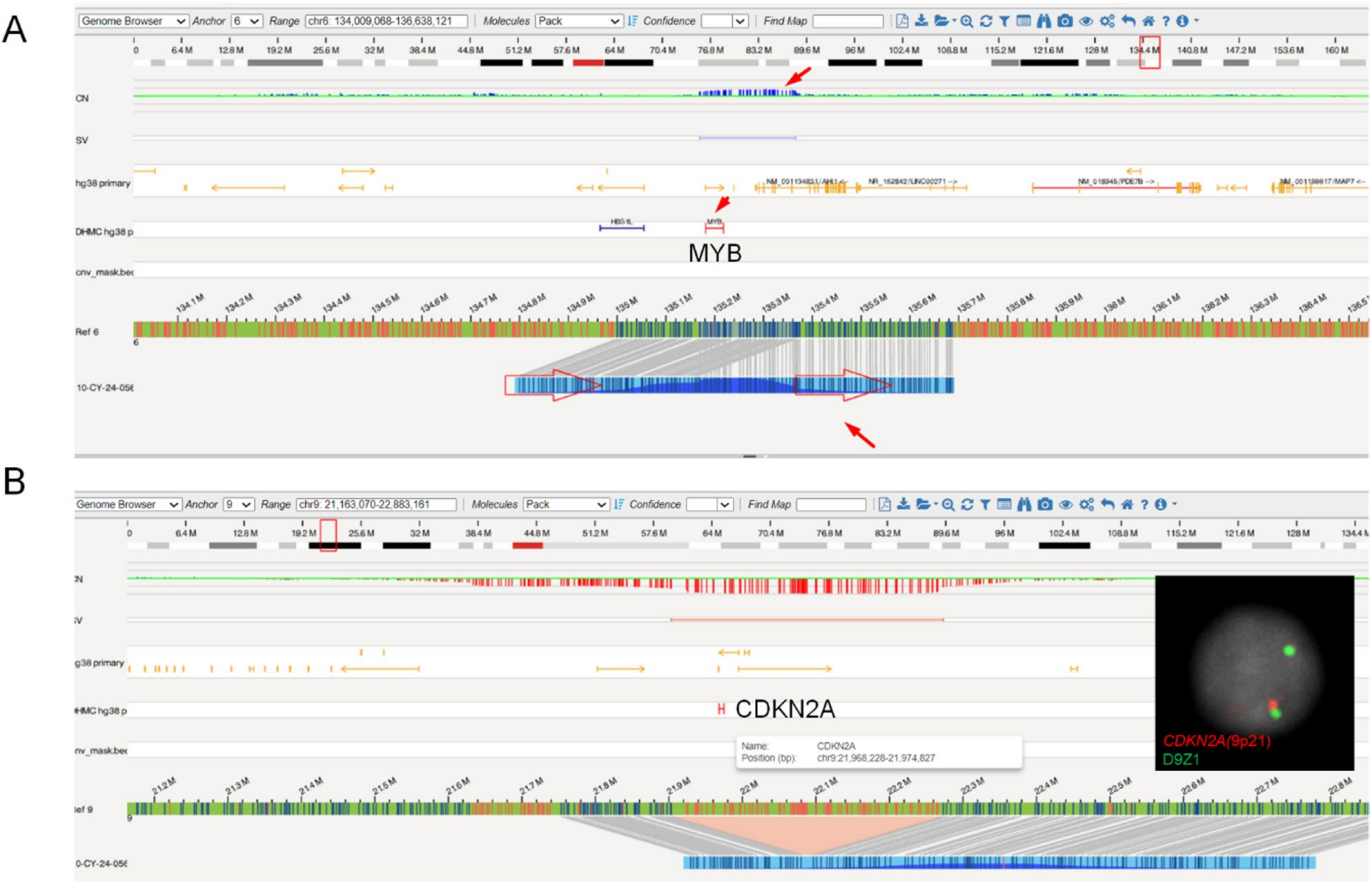
**(A)** Optical genome mapping detected a 197.1 kb duplication (ogm[GRCh38] dup [(6])(q23.3q23.3)(135168790_135365969) in chromosome 6q23.3 with a variant allele fraction of 0.360. This duplication includes the entire *MYB* gene. **(B)** A 353.3 kb heterozygous deletion (ogm[GRCh38] 9p21.3(21,903,070_22,273,254)x1) in chromosome 9p21.3 with a variant allele fraction of 0.610 shows loss of *CDKN2A*. Interphase FISH analysis confirms a one copy loss of *CDKN2A* (one spectrum red signal) with a centromere (D9Z1, two spectrum green signals) and locus specific DNA FISH probe. Nuclei are stained DAPI (inverse gray scale shown)

OGM also detected likely oncogenic variants implicated in T-ALL as emerging targets that are known aberrancies in other hematologic malignancies. This involved a rare 197.1 kb focal duplication of chromosome 6q23.3 encompassing the entire *MYB* gene (Table 1; Fig. 4A). Focal copy‑number gain involving *MYB* at 6q23.3 detected by OGM was also independently confirmed by exome‑based copy number analysis (Additional file 1: Figure S3). *MYB* is a transcription factor involved in T-cell development that is implicated in the pathogenesis and maintenance of B and T-cell neoplasms [17, 18]. *MYB* duplications have been detected in 5–8% of T-ALLs, but are more common in pediatric T-ALL compared to adult (5.2% vs. 2.8%) [19, 20]. Our detection of a *MYB* duplication with OGM adds to growing evidence that it likely plays a role in pathogenesis and maintenance of adult T-ALL. Further, a heterozygous 353.3 kb deletion including the *CDKN2A* gene was detected by OGM and further confirmed by FISH in approximately 87% of nuclei (Table 1; Fig. 4B). *CDKN2A* deletions have been detected in 23–55% of T-ALL cases [21, 22]. Multiple meta-analyses have shown that *CDKN2A* deletions are associated with lower event-free survival in adult T-ALL patients [21, 23, 24].

In light of the overall favorable risk profile, the patient was started on induction therapy using the AALL1231 regimen with bortezomib, which has been shown to improve survival in patients with T‑lymphoblastic leukemia [25]. At post-induction staging, the patient had a normal male karyotype.

## Discussion and conclusions

This case demonstrates a wide range of causative SVs, detected in the current study, that were not part of the initial cytogenetic and sequencing workup. Its ability to capture a large spectrum of variants in an unbiased manner makes a genome mapping approach especially useful for complex cases of T-ALL that can have multiple chromosomal rearrangements, thus aiding in the detection of disease-defining variants and determining prognostic classification. Not only has OGM been shown to be highly concordant with the standard-of-care workup for ALL, it has also detected additional variants with notable impact in several reported cases across a range of hematolymphoid malignancies [6, 7, 26, 27]. In a study by Toruner et al., OGM identified additional Tier I oncogenic and Tier II likely oncogenic variants in 90% of T-ALL cases, with *TLX3*::*BCL11B* rearrangements being the most common as compared to karyotyping and FISH [7]. Vieler et al. examined 11 adult B- and T-ALL cases using OGM and identified additional variants in 82% of cases [26]. In a study by Rack et al., OGM was able to identify the partner chromosome for 3 pediatric/adult T-ALL cases in which FISH identified a rearrangement on one chromosome but could not determine the partner, thus clarifying otherwise inconclusive results [6].

Some of the aberrations identified in this case map to lesions that have been associated with favorable or unfavorable outcomes as noted in Table 1, resulting in a mixed prognostic profile that can be difficult to interpret. Guidelines from the NCCN, ICC, and European LeukemiaNet provide a framework for integrating such discordant findings, but genomic risk categories are currently better validated in AML, MDS, and CLL cohorts with OGM than in T‑ALL. Inpractice, mixed‑risk T‑ALL cases are usually interpreted by prioritizing lesions with clearly established prognostic impact together with protocol‑defined clinical features, while additional OGM‑defined structural variant events are considered supportive or hypothesis‑generating rather than independently risk‑defining. For instance, in a study by Soler et al., OGM results were interpreted in the context of variants detected by standard-of-care methods, and risk stratification was based on variants with clearly defined prognostic value [28]. OGM also detected additional variants in a *BCR::ABL1*-positive B-ALL, including an *IZFK1* variant that is associated with resistance to tyrosine kinase inhibitors. While this did not change the risk stratification, these results may have guided treatment decisions [28]. Additionally, the International Consortium for OGM has released guidelines to aid in the integration of OGM in the logistic and prognostic work-up of hematologic malignancies [29].

In this T-ALL case, OGM also detected a hematopoietic tumor suppressor gene, *PHF6*, for which loss-of-function mutations occur in 38% of adult T-ALL cases [30]. *PHF6* variants are more likely to be associated with *NOTCH1* variants than wild-type *PHF6* [31]. *PHF6* loss-of-function variants are also associated with *TLX3* activating mutations, with a recent study showing that concurrent overexpression of *TLX3* in *PHF6* knockdown mice drove the development of early-onset leukemia [30]. The detection of both *PHF6* and *TLX3*::*BCL11B* variants in the current study contributes to the classification of this T-ALL and may point to clues regarding the pathogenesis of this subtype.

The detection of less-defined genetic targets in adult T-ALL by OGM points to the need for further investigation of their associated genetic aberrancies and their role in the pathogenesis of T-ALL to be clinically useful. Genes such as *CDKN2A* and *MYB* are affected in 23% and 2.8% of adult T-ALL cases, respectively, but their prognostic significance is less well-defined [20, 21]. Vieler et al. detected *CDKN2A* deletions in 7/11 patients with adult B- or T-ALL using OGM which, due to its higher resolution, revealed a wide variety of deletions that may aid in detection in the future [26]. In infant/toddler T-ALL, evaluation using OGM detected a correlation between *MYB* and *NKX2* rearrangements and a favorable prognosis in this group compared to other subgroups [32]. Thus far, only tandem duplications of *MYB* have been detected in adult T-ALL, but additional investigation may yield valuable information about the frequency of *MYB* alterations, its association with other variants, and prognostic significance [21]. OGM is a promising method to elucidate the genome-wide chromosomal structural architecture of T-ALL subtypes, which can help minimize reflexive testing, finetune prognosis, and potentially develop new therapeutic approaches.

Other genomic platforms could capture parts of the structural landscape observed in this T-ALL case. Exome sequencing with depth‑of‑coverage copy number variant analysis and RNA‑sequencing for fusion detection could, in principle, detect several lesions, including *FBXW7* and *MYB* copy‑number changes and *TLX3*::*BCL11B* fusion transcripts, when pipelines are optimized. However, regulatory rearrangements such as *TCR* enhancer–driven oncogene activation in T‑ALL, more broadly, and the insertional rearrangement, such as the *TLX3*::*BCL11B* identified here, are fundamentally DNA‑level events that may not consistently yield detectable or well‑annotated chimeric transcripts, limiting the sensitivity of RNA‑sequencing alone. Further, small intragenic deletions or duplications may pose challenges for reliable sequence depth-based copy number calling. OGM directly delineates these DNA breakpoints and their genome‑wide structural context, offering a complementary approach to resolving complex regulatory architecture.

In conclusion, the use of OGM in T-ALL provides a thorough assessment of underlying structural variants, facilitating the clarification of complex or inconclusive findings detected by conventional methods. Its capacity to reveal challenging rearrangements, such as the insertional translocation identified in the current study, as well as characterize less-defined targets, highlights opportunities for further study into their biological and prognostic implications, particularly in adult T-ALL where such correlations remain limited.

## Supplementary Information

**Supplementary material 1**: Figure S1. Exome-based copy number analysis demonstrating a 3.282 Mb deletion at chr4:152204259-155487161 (hg19) encompassing the *FBXW7* gene (black box). The CNV log2 ratio track shows loss of coverage, and the Copy Number Variants Segment track displays the deletion with reduced copy number state. Read-depth analysis confirms loss of *FBXW7*, a tumor suppressor frequently altered in T-cell acute lymphoblastic leukemia.

**Supplementary material 2**: Figure S2. Exome-based copy number analysis demonstrating a hemizygous loss at chrX:133371211-133380872 (hg19) affecting the *PHF6* gene (black box). The CNV log2 ratio shows decreased coverage consistent with deletion, while the Copy Number Variants Segment track confirms copy number loss. *PHF6* is an X-linked tumor suppressor gene recurrently deleted in hematologic malignancies, particularly T-ALL and AML.

**Supplementary material 3**: Figure S3. Exome-based copy number analysis demonstrating a 95 kb duplication at chr6:136172838-136268672 (hg19) involving the *MYB* gene (black box). The CNV log2 ratio track demonstrates increased coverage, and the Copy Number Variants Segment track displays the gain (red/maroon segment) with elevated copy number (black box). Focal *MYB* gain is a recurrent oncogenic driver in acute leukemia.

## Data Availability

All relevant data generated or analyzed during this study are included in this published article.

## Abbreviations

ALL: Acute lymphoblastic leukemia
OGM: Optical genome mapping
TdT: Terminal deoxynucleotidyl transferase
FISH: Fluorescence in situ hybridization
NGS: Next generation sequencing
SV: Structural variant
VAF: Variant allele fraction
