# Supplementary figures and images for: Optical genome mapping uncovers disease-defining variants in an adult T-lymphoblastic leukemia and impacts prognosis
Source: Mol Cytogenet. 2026 May 24;19:27. doi: 10.1186/s13039-026-00770-7 (PMC13377857; doi:10.1186/s13039-026-00770-7)

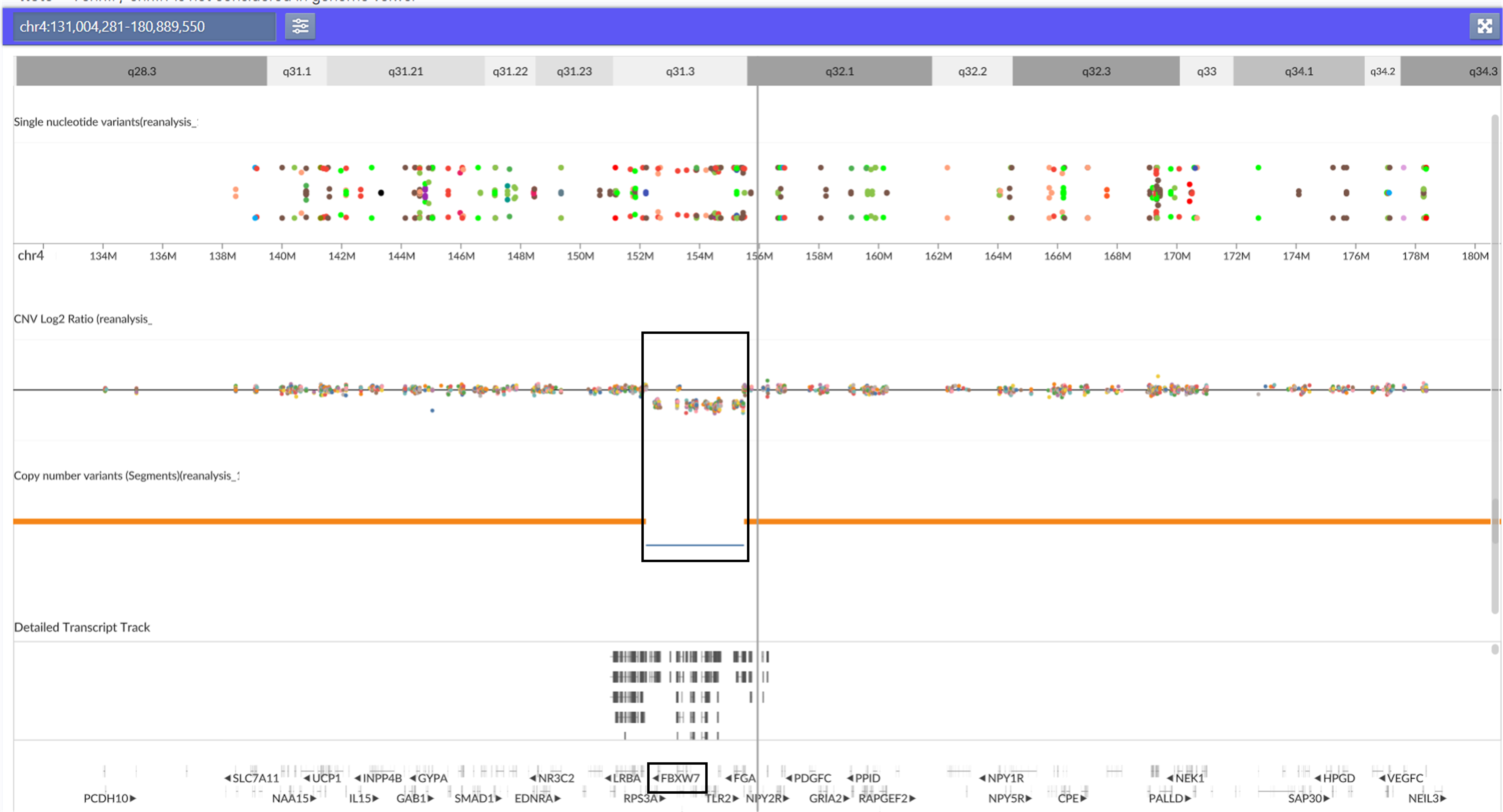

Supplement: Supplementary file 1 — Supplementary material 1: Figure S1. Exome-based copy number analysis demonstrating a 3.282 Mb deletion at chr4:152204259-155487161 (hg19) encompassing the FBXW7 gene (black box). The CNV log2 ratio track shows loss of coverage, and the Copy Number Variants Segment track displays the deletion with reduced copy number state. Read-depth analysis confirms loss of FBXW7, a tumor suppressor frequently altered in T-cell acute lymphoblastic leukemia. [file 13039_2026_770_MOESM1_ESM.tif]

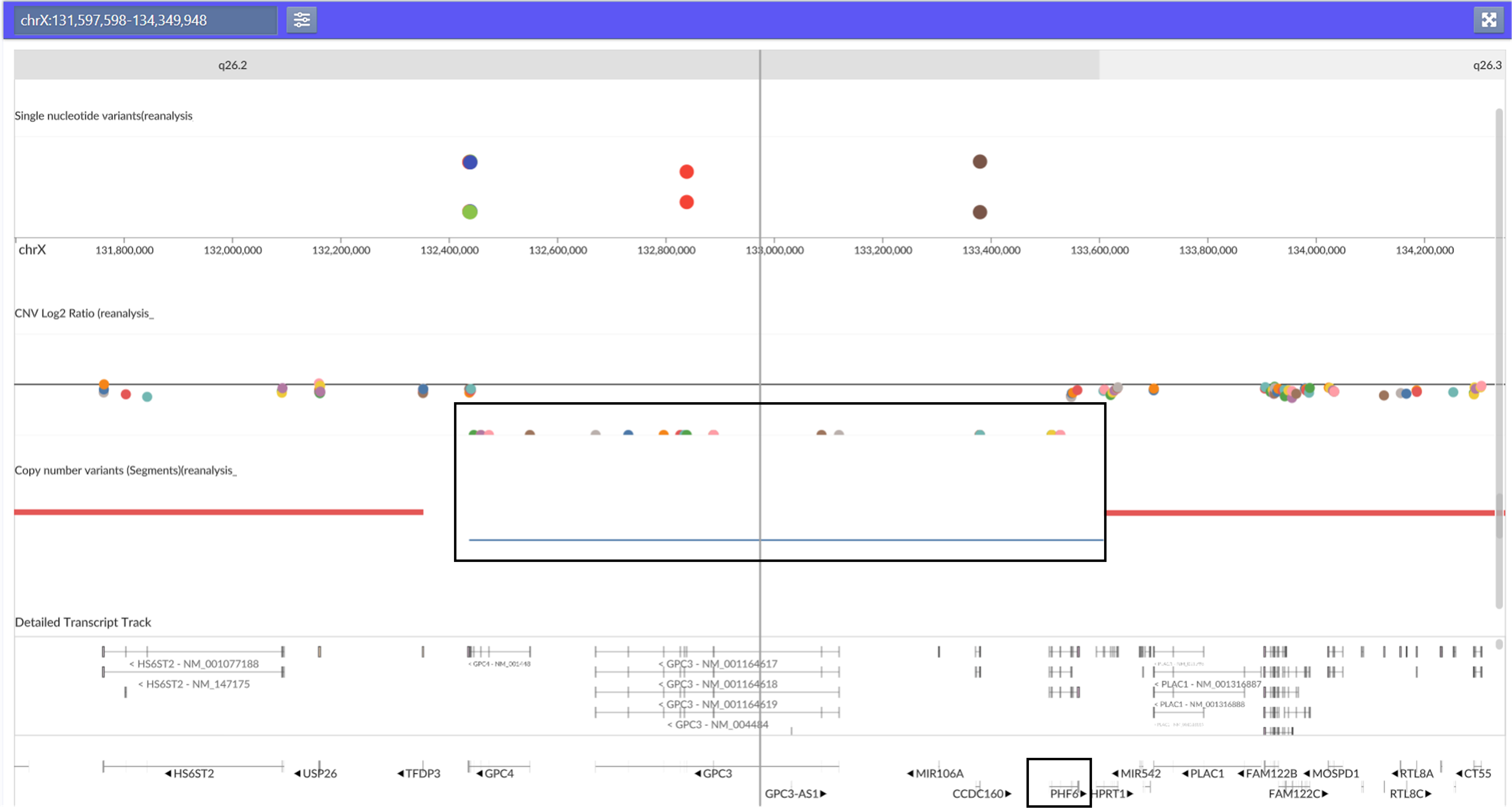

Supplement: Supplementary file 2 — Supplementary material 2: Figure S2. Exome-based copy number analysis demonstrating a hemizygous loss at chrX:133371211-133380872 (hg19) affecting the PHF6 gene (black box). The CNV log2 ratio shows decreased coverage consistent with deletion, while the Copy Number Variants Segment track confirms copy number loss. PHF6 is an X-linked tumor suppressor gene recurrently deleted in hematologic malignancies, particularly T-ALL and AML. [file 13039_2026_770_MOESM2_ESM.tif]

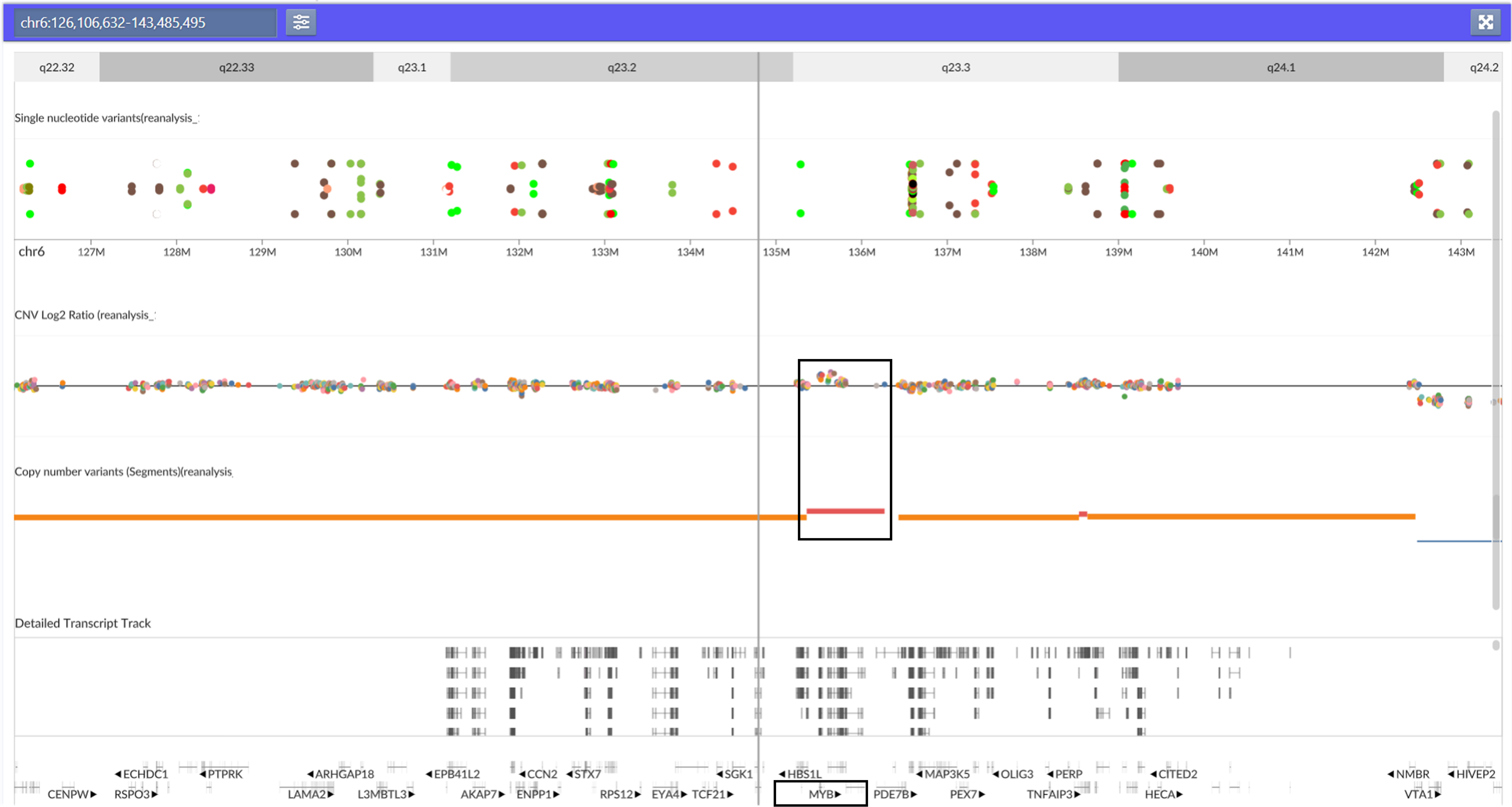

Supplement: Supplementary file 3 — Supplementary material 3: Figure S3. Exome-based copy number analysis demonstrating a 95 kb duplication at chr6:136172838-136268672 (hg19) involving the MYB gene (black box). The CNV log2 ratio track demonstrates increased coverage, and the Copy Number Variants Segment track displays the gain (red/maroon segment) with elevated copy number (black box). Focal MYB gain is a recurrent oncogenic driver in acute leukemia. [file 13039_2026_770_MOESM3_ESM.tif]
